# Stanniocalcin-1 inhibits thrombin-induced signaling and protects from bleomycin-induced lung injury

**DOI:** 10.1038/srep18117

**Published:** 2015-12-07

**Authors:** Luping Huang, Lin Zhang, Huiming Ju, Qingtian Li, Jenny Szu-Chin Pan, Zahraa Al-Lawati, David Sheikh-Hamad

**Affiliations:** 1 Division of Nephrology and Selzman Institute for Kidney Health/Department of Medicine, Baylor College of Medicine, Houston, TX, United States; 2 Center of General Surgery, Chengdu General Hospital of Chengdu Military Area Command, Chengdu, P.R. China; 3 College of Veterinary Medicine, Yangzhou University, Yangzhou 25009, Jiangsu, P.R.China

## Abstract

Thrombin-induced and proteinase-activated receptor 1 (PAR1)-mediated signaling increases ROS production, activates ERK, and promotes inflammation and fibroblast proliferation in bleomycin-induced lung injury. Stanniocalcin-1 (STC1) activates anti-oxidant pathways, inhibits inflammation and provides cytoprotection; hence, we hypothesized that STC1 will inhibit thrombin/PAR1 signaling and protect from bleomycin-induced pneumonitis. We determined thrombin level and activity, thrombin-induced PAR-1-mediated signaling, superoxide generation and lung pathology after intra-tracheal administration of bleomycin to WT and STC1 Tg mice. Lungs of bleomycin-treated WT mice display: severe pneumonitis; increased generation of superoxide; vascular leak; increased thrombin protein abundance and activity; activation of ERK; greater cytokine/chemokine release and infiltration with T-cells and macrophages. Lungs of STC1 Tg mice displayed none of the above changes. Mechanistic analysis in cultured pulmonary epithelial cells (A549) suggests that STC1 inhibits thrombin-induced and PAR1-mediated ERK activation through suppression of superoxide. In conclusion, STC1 blunts bleomycin-induced rise in thrombin protein and activity, diminishes thrombin-induced signaling through PAR1 to ERK, and inhibits bleomycin-induced pneumonitis. Moreover, our study identifies a new set of cytokines/chemokines, which play a role in the pathogenesis of bleomycin-induced lung injury. These findings broaden the array of potential therapeutic targets for the treatment of lung diseases characterized by thrombin activation, oxidant stress and inflammation.

Thoracic malignancies are among the leading cause of morbidity and mortality. Radiation and chemotherapy, commonly used for the treatment of thoracic malignancies, are frequently associated with pneumonitis and pulmonary fibrosis^1^. The pathogenesis of pulmonary fibrosis involves alveolar epithelial and endothelial cell injury, increased reactive oxygen species (ROS) and expression of cytokines/chemokines, inflammation, fibroblast activation and proliferation with consequent matrix deposition in the alveolar and interstitial spaces, leading to tissue damage, fibrosis, reduced lung volume and compliance^2^.

Thrombin is a multifunctional serine protease that catalyzes the conversion of fibrinogen to fibrin and plays an important role in blood coagulation. In addition, thrombin is involved in tissue repair, wound healing and lung fibrosis via activation of protease-activated receptors (PARs), a family of G protein–coupled receptors comprised of four members (PAR1–4)^3^,^4^. PAR1 has been identified as the major receptor for thrombin-induced mitogenic, inflammatory, and fibrotic effects^3^,^4^. There is considerable evidence to suggest important roles for thrombin, ROS and extracellular regulated kinase (ERK) activation in the pathogenesis of inflammation and immune-mediated lung injury^5^.

Mammalian STC1 is ubiquitously expressed and has been detected in many tissues including the lungs^6^. While it circulates in the blood, it is believed to function as an autocrine/intracrine substance^7^. Studies from our lab suggest that mammalian STC1 upregulates uncoupling proteins and suppresses mitochondrial superoxide generation^8^; and in doing so, it inhibits macrophage function^8^, attenuates cytokine-induced rise in endothelial permeability^9^ and migration of lymphocytes and macrophages across endothelial cells^10^. Combined, these effects predict potent anti-inflammatory action. Indeed, STC1 Tg mice are protected from ischemia/reperfusion kidney injury^11^ and anti-glomerular basement membrane (GBM) glomerulonephritis (GN)^12^; models of kidney injury involving ROS and inflammation. Of note, protection from anti-GBM glomerulonephritis in STC1 Tg mice, is associated with diminished expression of macrophage chemotaxis protein-1 (MCP-1), transforming growth factor-β (TGF-β) and MIP2 in the kidney^12^. Based on our observations, we hypothesized that transgenic overexpression of STC1 in mice will inhibit thrombin actions, diminish ROS production and inflammation, and protect from bleomycin-induced lung injury and inflammation.

Our data reveal novel effects by STC1: transgenic overexpression of STC1 diminishes thrombin protein abundance and activity; decreases superoxide generation; down-regulates ERK activity; decrease cytokines/chemokines release; and reduces vascular permeability and accumulation of inflammatory cells in the lungs after bleomycin administration. Mechanistic *in vitro* data suggest that STC1 inhibits thrombin/PAR1-mediated signaling to ERK through suppression of superoxide. Our findings are clinically relevant and may provide new therapeutic targets for the treatment/prevention of radiation and chemotherapy induced-pneumonitis, and consequent pulmonary fibrosis.

## Materials and Methods

### Materials

All materials were purchased from Sigma Aldrich Inc. (St Louis, MO) unless stated otherwise. Recombinant human STC1 (rSTC1) was purchased from MyBioSource (San Diego, CA). MnTBAP, rat anti-F4/80 (macrophage marker), rabbit anti-CD3 (T-cell marker), mouse anti-actin and mouse anti-GAPDH were purchased from EMD Millipore (Billerica, MA). SCH79797 was purchased from Fisher Scientific (Houston, TX). Goat anti-hSTC1 antibodies and Goat anti-thrombin antibodies were purchased from Santa Cruz (Santa Cruz, CA). Rabbit anti-ERK and rabbit anti-p-ERK were purchased from Cell Signaling (Danvers, MA).

### Mice

STC1 Tg mice on C57B/6 genetic background were generated by Varghese *et al.*^13^ and were further characterized and described elsewhere^11^,^12^. All studies were carried out using mice homozygous for the transgene (derived from line 2^13^), and WT generated from crosses between heterozygous mice. Transgenic mice display elevated rSTC1 in the serum and preferential expression of the transgene in the heart, liver, brain, testis, mammary glands and adipose tissue^13^. Mice were maintained in air conditioned rooms under pathogen-free conditions with 12 h/12 h light/dark cycles, and were given free access to food and water. The investigation conforms to the Guide for the Care and Use of Laboratory Animals published by the US National Institutes of Health, and the experiments were approved by Baylor College of Medicine Institutional Animal Care and Use Committee (IACUC).

### Mouse model of bleomycin-induced lung injury

Age-matched WT and STC1 Tg mice were used. Mice were anesthetized and treated with single-dose of saline or bleomycin (2 U/kg; in a total volume of 50 μl of saline) via intra-tracheal injection as previously described^14^; 6–10 mice were used for each group at each time point.

### Immunohistochemistry and morphology

Whole lungs were removed, fixed by Methanol-Carnoy (Methacarn), paraffin-embedded, and 5-μm sections were obtained. Lung tissue was stained with: H&E (morphology); anti-F4/80 (macrophages); anti-CD3 (lymphocytes). Detection was carried out using peroxidase enzyme-based detection system (Vector Laboratories), and photomicrographs were taken using Nikon Eclipse 80i microscope system. Neutrophils were identified based on morphology and counted. The severity of lung injury was determined as published by Li *et al.*^15^, and grading was based on the degree of inflammation and extent of lung injury: grade 0, normal tissue; grade 1, <20% of the surveyed tissue is injured and mild inflammation; grade 2, 20–50% of the surveyed tissue is injured and moderate inflammation; and grade 3, >50% of the surveyed tissue is injured with severe inflammation. The mean score from all examined fields was calculated as the injury/inflammation score (IS).

### Measurement of pulmonary vascular permeability

Vascular permeability was measured using a modification of a previously described method^16^. Briefly, Evans blue dye (20 mg/kg body weight) prepared in PBS was injected through the tail vein. Mice were sacrificed after 1 hour, the lung vasculature was perfused with PBS; the lungs were excised, placed in 2 mL of formamide, and homogenized. The homogenates were incubated at 60 °C for 16 hours, followed by centrifugation at 8000 g for 10 min, and the supernatants were collected. Absorbance of the supernatants was measured at 620 nm and normalized to the absorbance of wells containing assay buffer alone, giving permeability index (expressed as OD/mg protein).

### Thrombin activity assay

Lung tissue was rinsed in PBS, and homogenized in 500 μL of PBS, followed by centrifugation at 3000 g for 5 min, at 4 °C; the supernatant was collected and the protein was quantitated (Bradford’s method). Tissue lysates (100 μg protein/100 μL) were placed in black wall 96-well flat-bottom plates, and assayed using SensoLyte® 520 thrombin activity assay kit (AnaSpec, Inc.; Fremont, CA) as per manufacturer’s instructions. This kit contains a novel internally quenched 5-FAM/QXL^®^ 520 fluorescence resonance energy transfer (FRET) substrate for thrombin. Cleavage of the FRET substrate by thrombin separates the fragments, releasing 5-FAM fluorescence (monitored at excitation/emission = 490 nm/520 nm).

### Superoxide measurements

Superoxide was measured using a modification of a previously described method^11^. Briefly, lung tissue was homogenized in 500 μL sucrose buffer (composition: 0.31 M sucrose; 10 mM Tris-HCl; pH 7.4) on ice, followed by protein quantitation (Bradford’s method). Tissue lysates (100 μg protein/100 μL) were placed in clear flat-bottom wells (96-well plate), containing excess dihydroethidium (DHE; 100 μM), in a final volume of 100 μL/well. Absorbance values (excitation/emission at 530/620 nm) were normalized to the absorbance of wells containing equimolar concentrations of DHE alone, and expressed as OD/μg protein.

### Cell culture

Human basal alveolar epithelial cells (A549 cells) were cultured in Dulbecco’s Modified Eagle’s medium (DMEM) containing 10% fetal bovine serum (FBS) and 100 U/mL of each penicillin and streptomycin, and maintained in an incubator set at 37 °C and containing humidified atmosphere of 5% CO_2_ in air. At 80% cell confluence, the growth medium was replaced with DMEM containing 1% FBS and the cells were weaned overnight. The following day, the medium was replaced with 1 mL of DMEM devoid of FBS or growth factors, and cells were treated with one of the following: thrombin (1 U/mL); rSTC1 (200 ng/mL); SCH79797 (a PAR1 antagonist; 4 μM); MnTBAP (antioxidant; 0.1 mM); paraquat (a superoxide generator; 40 μM); or combination of the above. Cells were collected, assayed for MitoSOX (Invitrogen, Carlsbad, CA) fluorescence, or lysed in radioimmunoprecipitation assay buffer [RIPA; composition: 150 mM NaCl; 50 mM Tris-HCl (pH 7.4); 1% NP-40; 0.25% sodium deoxycholate; 1 mM ethylene diamine tetra acetic acid (EDTA)] containing complete mini protease inhibitor (Roche Applied Science, Indianapolis, IN), and phosphatase inhibitor cocktail (Sigma) for Western blot analysis. MitoSOX permeates live cells and selectively targets the mitochondria; it is rapidly oxidized by superoxide and emits red fluorescence.

### MitoSOX fluorescence assay

Measurement of superoxide in cells was performed as previously described^11^. Briefly, cells were seeded onto coverslips placed at the bottom of 6-well plates and treated as detailed above. MitoSOX™ and bis Benzimide 33342 trihydrochloride (Hoechst; Sigma; used for counter stain) were added to the culture media for 10 min, at a final concentrations of 5 μM and 0.2 μg/mL, respectively. Following incubation with MitoSOX and Hoechst, cells were rinsed in PBS and fluorescence was visualized (excitation/emission at 510/580 nm).

### Western blotting

Lysates, prepared from A549 cells or whole lung tissue as previously described^11^, were suspended in RIPA buffer containing 1x cocktail of proteinase and phosphatase inhibitors, and centrifuged at 8000 g, 4 °C for 10 min, to remove cell debris. Twenty μg of protein were resolved on 12% SDS-PAGE, transferred to nitrocellulose membrane and incubated with primary antibodies for: STC1; ERK; p-ERK; thrombin (also recognizes prothrombin); actin or GAPDH. After wash with PBS containing 0.1% Tween-20, the membrane was incubated with horseradish peroxidase-conjugated secondary antibody. The bound antibodies were visualized using chemiluminescence. Densitometric analysis of target proteins was performed using NIH ImageJ Software. The activity of ERK was determined based on the ratio of p-ERK/t-ERK bands.

### Cytokine polymerase chain reaction (PCR) array

Mouse Common Cytokines RT^2^ Profiler™ PCR Array (QIAGEN; Frederick, MD), employing a set of optimized PCR primers was used to profile the expression of 84 important cytokine genes using real-time (RT)-PCR CFX 96 system (Bio-Rad; Hercules, CA). This array includes tumor necrosis factor-α (TNF-α), interferons, interleukins, bone morphogenic proteins (BMP) and members of the TGF-β family. Also represented are various growth factors (colony-stimulating, fibroblast, insulin-like, platelet-derived, transforming, and vascular endothelial). Total RNA was isolated from whole lungs, using TRIzol (Invitrogen; Grand Island, NY). RNAs were treated with DNase I prior to cDNA synthesis, and 150 ng of total RNA were reverse-transcribed into cDNA using the RT2 First Stand Kit (QIAGEN, Frederick, MD) as per manufacturer’s instructions. Relative mRNA expression levels were calculated using RT^2^ Profiler Array Data Analysis software v3.5 (QIAGEN). Gene expression levels were normalized to five housekeeping genes included in the Array. Fold changes in gene expression were calculated by the software using the 2-∆∆Ct method. 2-fold or greater change in gene expression was set as the threshold for a meaningful change in gene expression. Data represent the mean± SEM of three independent determinations.

### ELISA

We used an enzyme-linked immunosorbent assay (ELISA) to determine the levels of Spp1 and CXCL16 proteins in lung tissue. Briefly, MaxiSorp (Nunc) plate wells were coated with anti-Spp1 or anti-CXCL16 antibodies (50 μL/well) at 37 °C for 2–3 hours, followed by wash in PBS (6 times); the wells were then blocked with 1% BSA in PBS for 1 hour, washed again in PBS (6 times) before the addition of equal amounts of protein lysates, followed by gentle agitation at RT for 2 h; lysis buffer was used as negative control. Next, biotinylated anti-CXCL16 or anti-Spp1 (50 μl/well) was added and incubated for 1 hour; wells were then washed in PBS (6 times), followed by the addition of poly-HRP streptavidin (50 μl/well) and incubation in the dark for 30 additional minutes. Following wash in PBS (6 times), freshly prepared TMB-peroxidase solution (100 uL/well) was added for 2–5 min, and the reaction was stopped using 1 N H_2_SO_4_. Signal intensity was determined using ELISA reader at A_450_ nm.

### Statistics

Statistical analysis was performed using Graph Pad Prism Software version 4. Differences between data groups were evaluated for significance using unpaired t-test, one way ANOVA or Newman-Keuls for multiple comparisons. Statistical significance was defined by a *p < *0.05.

## Results

### STC1 protects from acute bleomycin-induced pneumonitis

**Figure 1:**
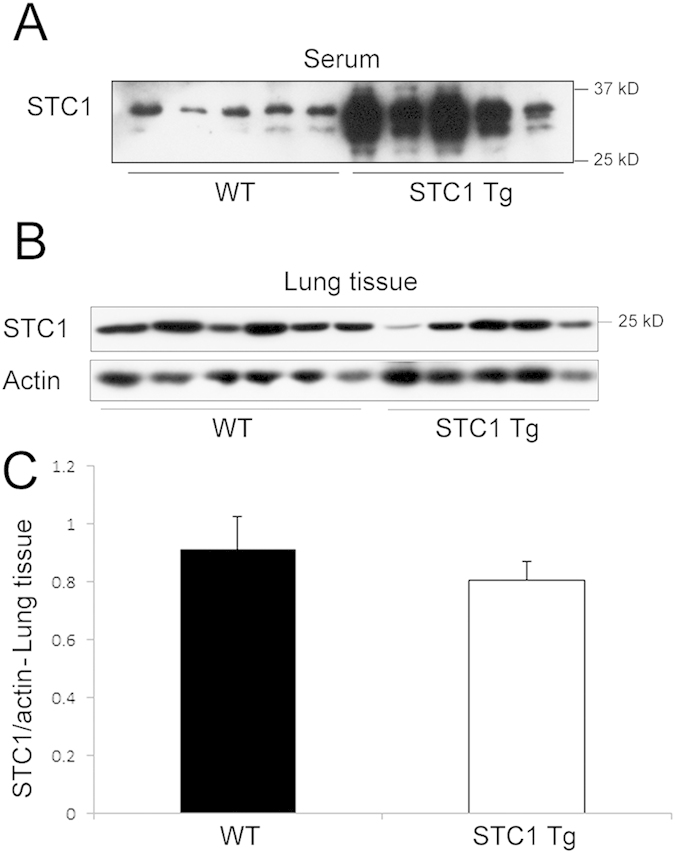
Lung tissue and serum levels of STC1 in WT and STC1 Tg mice. (**A**) Equal amounts of serum proteins from WT and STC1 Tg mice were run on SDS-PAGE, and Western blot was reacted with anti-STC1 antibodies. (**B**) Lung tissue lysates were reacted with anti-STC1 or anti-actin; representative blots are shown. (**C**) STC1 band intensities were normalized to actin bands, and bar graph depicts the mean± SEM (n = 5).

**Figure 2:**
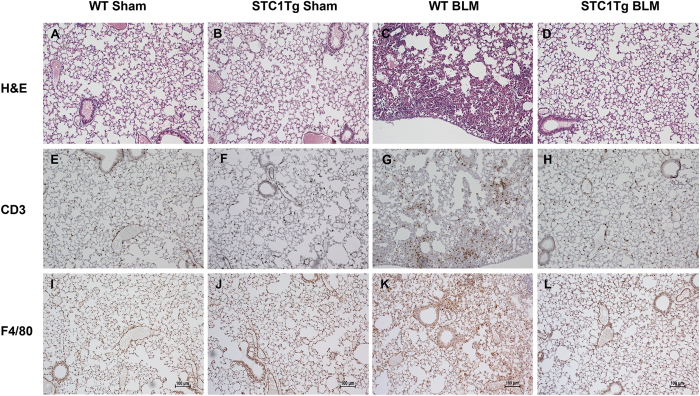
Transgenic overexpression of STC1 inhibits bleomycin-induced pneumonitis. Wild type (WT) or STC1 transgenic (Tg) mice were treated with either saline or bleomycin (BLM). Lung tissue was harvested after one week, and subjected to immunohistochemistry. Top panels, H&E staining for morphology. Middle panels, show staining with anti-CD3 (lymphocytes). Bottom panels show staining with anti-F4/80 (macrophages).

**Figure 3:**
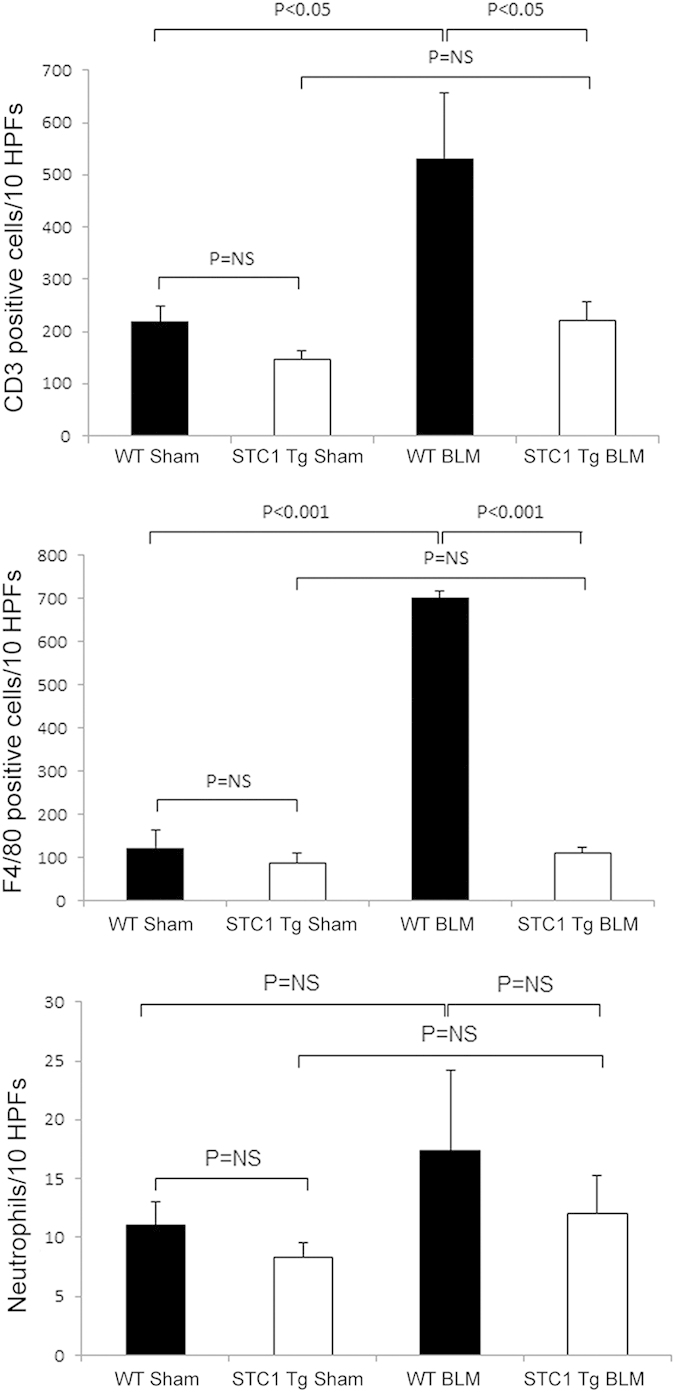
Transgenic overexpression of STC1 diminishes lung infiltration with macrophages, T-cells and neutrophils after bleomycin treatment. Wild type (WT) or STC1 transgenic (Tg) mice were treated with saline or bleomycin (BLM). Lung tissue was harvested after one week and stained with H&E for neutrophil count, anti-CD3 (lymphocytes), or anti-F4/80 (macrophages). Bar graphs show count of macrophages, T-cells and neutrophils (identified based on morphology), and represent the mean ± SEM (n = 6).

We applied a well-established bleomycin-induced lung injury model to WT and STC1 Tg mice. Compared with WT mice, STC1 Tg mice display elevated serum levels of STC1 protein; but equal levels of STC1 in lung tissues (Fig. 1). Figure 2A,B show normal lung morphology in saline-treated (sham) WT and STC1 Tg mice, respectively. One week after treatment with bleomycin (BLM), WT lungs showed interstitial edema, severe distortion of lung morphology (Fig. 2C and Table 1), and marked infiltration with lymphocytes (Figs 2G and 3), macrophages (Figs 2K and 3) and neutrophils (Fig. 3). On the other hand, lungs of bleomycin-treated STC1 Tg mice displayed normal morphology (Fig. 2D and Table 1) that was indistinguishable from the morphology of saline-treated WT or STC1 Tg mice (Table 1), and revealed fewer lymphocytes (Figs 2H and 3), macrophages (Figs 2L and 3) and neutrophils (Fig. 3). The data suggest that transgenic overexpression of STC1 protects from acute bleomycin-induced lung injury and pneumonitis.

### Transgenic overexpression of STC1 maintains vascular barrier function and inhibits bleomycin-induced lung inflammation

**Figure 4:**
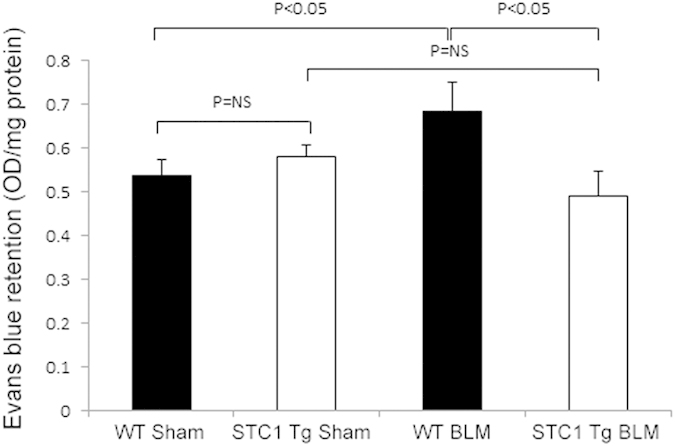
Transgenic overexpression of STC1 preserves vascular barrier function in the lung following bleomycin treatment. Evans blue dye was injected through the tail vein of WT and STC1 Tg mice one week after saline or BLM treatment. Lung tissue was harvested, homogenized; and the absorbance of Evans blue dye was measured at 620 nm. Values represent means ± SEM (n = 5).

In the current experiment, we examined vascular permeability across the pulmonary vasculature (measured as tissue retention of Evans blue) one week after treatment of WT and STC1 Tg mice with bleomycin. While vascular permeability increased in bleomycin-treated WT mice, we observed no changes in pulmonary vascular permeability of bleomycin-treated STC1 Tg mice (Fig. 4). These finding are consistent with preservation of endothelial barrier function by STC1, an effect that may contribute to the paucity of inflammatory cells we observe in the lungs of STC1 Tg mice after bleomycin treatment.

### Transgenic overexpression of STC1 diminishes thrombin level

**Figure 5:**
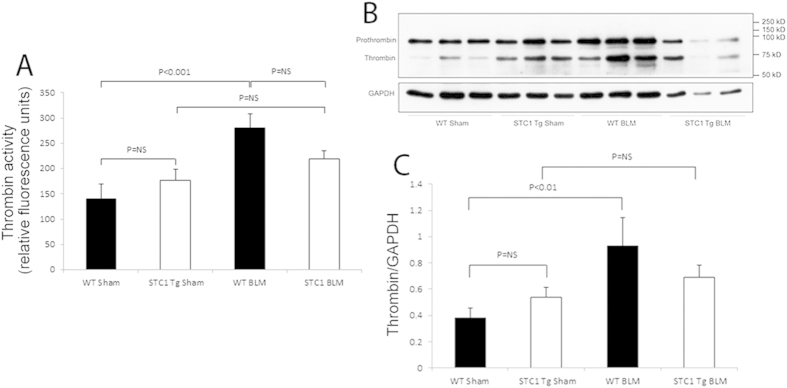
Transgenic overexpression of STC1 diminishes thrombin protein and activity in the lung after bleomycin treatment. One week after intra-tracheal administration of bleomycin, lungs were harvested, homogenized, and assayed for thrombin protein abundance and activity as described in methods. (**A**) The proteolytic activity of thrombin is expressed in relative fluorescent units. Values represent means ± SEM (n = 5). (**B,C**) Transgenic overexpression of STC1 diminishes thrombin protein expression. WT or STC1 Tg mice were treated with either saline or BLM. One week later, lungs were harvested, homogenized, protein lysates were resolved on SDS-PAGE, and Western blots reacted with anti-thrombin or anti-GAPDH. (**B**) Representative blots are shown. (**C**) Bar graph depicts band intensities for thrombin normalized to GAPDH; data represent the mean± SEM (n = 6–10).

Tissue injury elicits immediate responses that activate the coagulation cascade and lead to thrombin generation^17^, which has been shown to play an important role in lung inflammation and tissue repair^18^. Thrombin levels are markedly elevated in humans and experimental models of lung fibrosis^19^,^20^; hence, we examined thrombin level and activity in the lungs of WT and STC1 Tg mice after treatment with bleomycin. Compared with saline, treatment with bleomycin increased thrombin protein level and activity in the lungs of WT mice; and the increase in thrombin activity matches the increase in thrombin protein abundance (Fig. 5). However, treatment of STC1 Tg mice with bleomycin did not increase thrombin protein level or activity in the lungs beyond the baseline levels observed in saline-treated STC1 Tg mice. These data suggest that inhibition of thrombin in STC1 Tg mice may underlie (at least in part) the protection from bleomycin-induced lung injury.

### Suppression of superoxide by STC1 may contribute to the protection from bleomycin-induced lung injury

**Figure 6:**
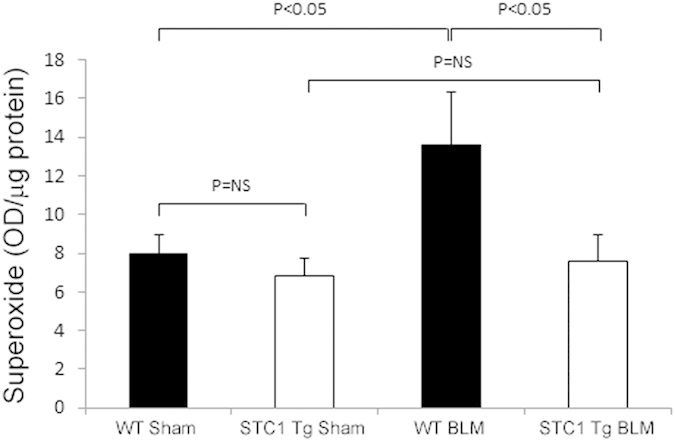
Transgenic overexpression of STC1 inhibits ROS production in the lungs after bleomycin treatment. One week after intra-tracheal administration of saline or BLM, lungs were harvested, homogenized, incubated with DHE, and fluorescence intensity was measured at excitation 530 nm, emission 620 nm. Values represent means ± SEM (n = 5).

Published data from our lab show STC1 suppresses superoxide generation in macrophages^8^ and cardiomyocytes^21^ via UCPs-dependent pathway. ROS play an important role in the pathogenesis of inflammation following bleomycin-induced lung injury^22^, and in the current experiment we sought to determine superoxide levels in the lungs of bleomycin treated mice. Our data reveal increased superoxide levels in WT lungs 1 week after treatment with bleomycin (Fig. 6), but not at 4 weeks (data not shown). On the other hand, superoxide generation was unchanged in STC1 Tg lungs after bleomycin treatment, and was indistinguishable from superoxide levels we observe in the lungs of saline-treated WT or STC1 Tg mice. Suppression of superoxide generation in STC1 Tg lungs after bleomycin treatment is likely related to decreased inflammation as shown above, as well as direct effects by STC1 on lung tissue; these results are in agreement with previously reported actions of STC1 to suppress superoxide generation in other tissues^8^,^21^ and may provide another mechanism for inhibition of bleomycin-induced lung injury.

### STC1 inhibits bleomycin-induced ERK1/2 activation *in vivo*

**Figure 7:**
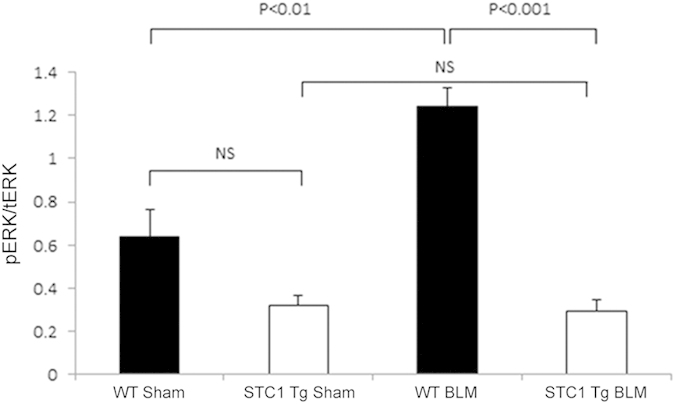
Transgenic overexpression of STC1 inhibits belomycin-induced ERK1/2 phosphorylation. WT or STC1 Tg mice were treated with either saline or BLM. One week later, lungs were harvested, homogenized, protein lysates were resolved on SDS-PAGE, and Western blots reacted with anti-ERK1/2 or anti-pERK1/2. Bar graph shows band intensities for pERK1/2 normalized to tERK1/2; data represent the mean± SEM (n = 6–10).

PAR1-mediated activation of PKC and ERK1/2 plays a critical role in thrombin-induced mitogenic, inflammatory, and pro-fibrotic effects^4^,^23^,^24^,^25^. ERK1/2 activation by thrombin follows PKC activation^25^, and in the current experiments, we sought to determine ERK1/2 activity (pERK1/2/tERK1/2) in WT and STC1 Tg lungs 1 week after saline (sham) or bleomycin (BLM) treatment. ERK1/2 activity was significantly increased in the lungs of WT mice after bleomycin treatment. ERK1/2 activity in the lungs of saline-treated STC1 Tg mice was numerically lower compared with saline-treated WT mice and was unchanged after blemoycin treatment (Fig. 7). Thus, STC1 inhibits baseline ERK1/2 activity, and blunts activation of ERK1/2 upon treatment with bleomycin.

### Blunted expression of pro-inflammatory cytokines/chemokines in STC1 Tg lungs after belomycin treatment

**Figure 8:**
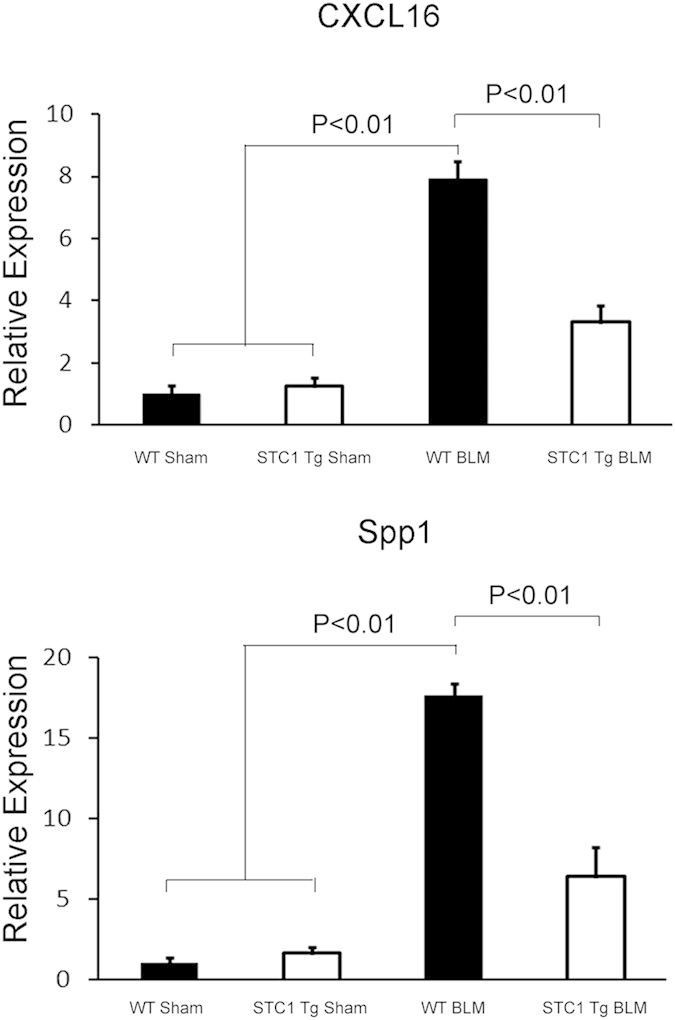
Transgenic overexpression of STC1 inhibits bleomycin-induced expression of CXCL16 and Spp1. WT or STC1 Tg mice were treated with either saline or BLM for one week. Lungs were harvested, homogenized, protein lysates were assayed for CXCL16 and Spp1 using ELISA. Bar graphs depict the means+ SEM (n = 3).

Thrombin-induced activation of ERK1/2 stimulates the expression of pro-inflammatory cytokines/chemokines and pro-fibrotic growth factors including: TNF-α; interleukin-6 (IL-6); IL-8; IL-13; IL-21; IL-1β; MCP-1; MIP-1; TGF-β; PDGF and bFGF^26^,^27^. Mouse Common Cytokines RT^2^ Profiler™ PCR Array was used to profile the expression of 84 important cytokine/chemokines and growth factors genes (see methods) in WT and STC1 Tg lungs after bleomycin treatment. As shown in Table 2, and compared with lungs of saline-treated WT mice, lungs of bleomycin-treated WT mice displayed significantly higher expression of: C-C chemokine ligand 17 (Ccl17; 6.7-fold); C-C chemokine ligand 2 (Ccl2; 6.7-fold); C-C chemokine ligand 7 (Ccl7; 8-fold); C-X-C chemokine 10 (Cxcl10; 4.6-fold); C-X-C chemokine 16 (Cxcl16; 5.1-fold); IL18 (3-fold); interleukin 1 receptor antagonist (IL1rn; 3.5-fold); leukemia inhibitory factor (Lif; 3.1-fold); secreted phosphoprotein 1 (Spp1; 19-fold) and beta-glucuronidase (Gusb; 2.3-fold); while IL5 was under-expressed (-2.7-fold). Cytokine/chemokine profile in the lungs of bleomycin-treated STC1 Tg mice was not different from the profile observed in saline-treated STC1 Tg mice (data not shown). Comparing the profile of cytokines/chemokines in the lungs of bleomycin-treated STC1 Tg mice with that of bleomycin-treated WT mice, we observe significantly lower expression of Ccl17 (-2-fold), chemokine (C-C motif) ligand 19 (Ccl19; -2-fold), Ccl2 (-3-fold), Ccl7 (-4-fold), Cxcl10 (-2.3-fold), Cxcl16 (-2.9-fold) and Spp1 (-6-fold) (Table 3); these constitute 7 out of 10 bleomycin-induced cytokines/chemokines in WT mice (Table 2). To validate the cytokine PCR array data, we determined protein levels of CXCL16 and Spp1 in lung tissue, using ELISA, and found increased expression of these proteins in the lungs of bleomycin-treated WT mice, but not in the lungs of bleomycin-treated STC1 Tg mice (Fig. 8). Our data identify new and previously unrecognized cytokines/chemokines that appear to play a role in the pathogenesis of bleomycin-induced acute lung injury; transgenic overexpression of STC1 blunts the expression of these cytokines/chemokines.

### STC1 inhibits bleomycin-induced thrombin/PAR1/ERK1/2 signaling *in vitro* through suppression of superoxide

**Figure 9:**
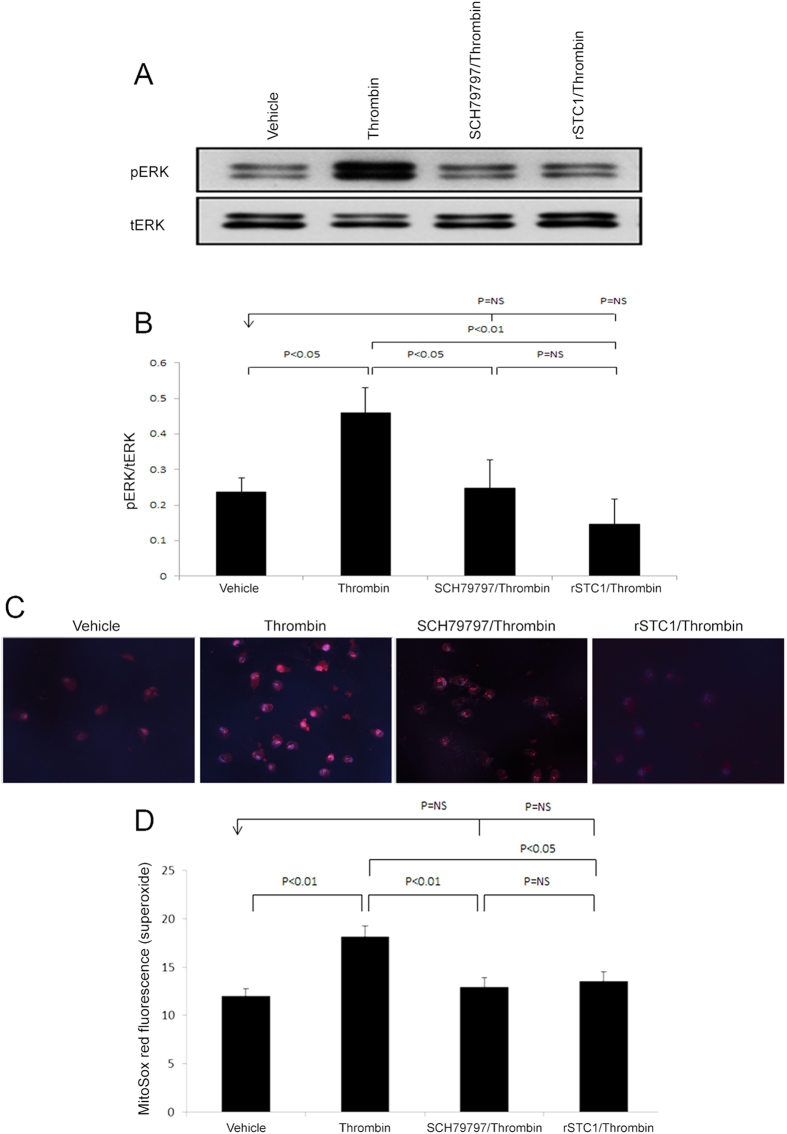
Recombinant STC1 (rSTC1) inhibits thrombin-induced and PAR1-mediated ERK1/2 activation and superoxide production. A549 cells were pre-treated with SCH79797 (4 μM) for 30 min or STC1 (200 ng/ml) for 2 h, followed by thrombin treatment (1 U/ml) for 10 min at 37 °C. Cells were harvested, lysed and proteins were resolved on SDS-PAGE. Western blots were reacted with anti-ERK1/2 or anti-pERK1/2. (**A**) Representative blots are shown. (**B**) Quantitation of pERK1/2 band intensities normalized to total ERK1/2 bands; bar graph depicts the mean ± SEM (n = 3). Thrombin induces superoxide through PAR1; recombinant STC1 attenuates thrombin-induced superoxide generation. A549 cells were treated as above, incubated with MitoSOX (5 μM) and Hoechst (0.2 μg/ml), and MitoSOX fluorescence was visualized (excitation 510 nm, emission 580 nm). (**C**) Representative images are shown; magnification: 200X. (**D**) Quantitation of MitoSOX fluorescence in A549 cells treated as above; bar graph depicts the mean± SEM (n = 3).

Our *in vivo* data suggest that transgenic overexpression of STC1 diminishes thrombin protein and activity, superoxide generation, ERK1/2 activation and release of cytokines/chemokines in the lungs after bleomycin treatment. To further characterize mechanisms of STC1 action, we utilized A549 pulmonary epithelial cells, which express high level of PAR1^28^ (the mediator of thrombin signaling), for *in vitro* analysis of the effects of thrombin and rSTC1. Thrombin activated ERK1/2 through PAR1, as the effect of thrombin was abolished by PAR1 specific inhibitor SCH79797 (Figs 9A and 7B)^29^. Similarly, STC1 inhibited thrombin-induced ERK1/2 activation (Fig. 9A). These data suggest inhibition of thrombin/PAR1 signaling by STC1 at the cellular level. We next examined the effect of STC1 on thrombin-induced superoxide generation. As shown in Fig. 9C,D, thrombin treatment increased mitochondrial superoxide generation markedly, an effect that was diminished by either SCH79797 or rSTC1, consistent with STC1-mediated inhibition of thrombin/PAR1-induced superoxide generation.

**Figure 10:**
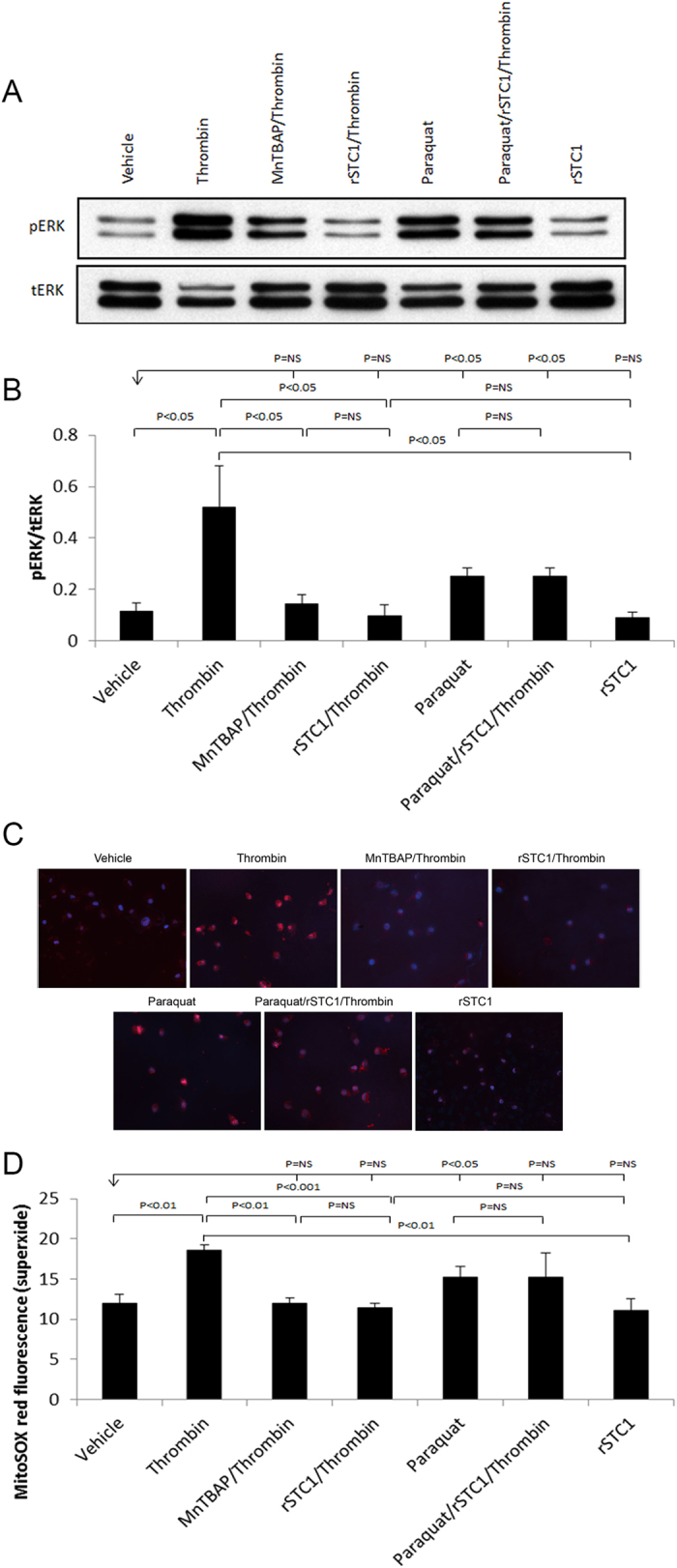
Recombinant STC1 inhibits thrombin-mediated ERK1/2 activation through suppression of superoxide production. A549 cells were pre-treated with the following agents, either singly, or in combination, followed by thrombin treatment (1 U/mL) for 10 min at 37 °C: paraquat (40 μM) for 4 h; MnTBAP (0.1 mM) for 30 min; STC1 (200 ng/mL) for 2 h. Cells were harvested, lysed, and proteins were resolved on SDS-PAGE; Western blots were reacted with anti-ERK1/2 or anti-pERK1/2. (**A**) Representative blots are shown. (**B**) Quantitation of pERK1/2 band intensities normalized to total ERK1/2 bands; bar graph depicts the mean + SEM (n = 3). (**C**) A549 cells, treated as above were incubated with MitoSOX (5 μM) and Hoechst (0.2 μg/ml) for 10 min, and MitoSOX fluorescence was visualized (excitation 510 nm, emission 580 nm). Representative images are shown; magnification: 200X. (**D**) Quantitation of MitoSOX fluorescence in A549 cells treated as above; bar graph depicts the mean± SEM (n = 3).

ERK1/2 activity is redox-sensitive^30^, and since STC1 suppresses superoxide generation, we examined the hypothesis that STC1 inhibits thrombin-induced ERK1/2 activation through suppression of superoxide. Indeed, while either thrombin or paraquat (a superoxide generator) induced ERK1/2 activation, MnTBAP [a cell permeable superoxide dismutase (SOD) mimic] or STC1 inhibited thrombin-induced ERK1/2 activation (Fig. 10). However, STC1 failed to block thrombin-induced ERK1/2 activation in the presence of paraquat (a superoxide generator). These data suggest that STC1 inhibits thrombin-induced ERK1/2 activation through suppression of superoxide generation.

## Discussion

Cumulative evidence suggests that thrombin promotes inflammation and fibroblast proliferation following bleomycin- and radiation-induced lung injuries^19^,^20^. PAR1 is expressed on epithelial cells, monocytes/macrophages and vascular endothelial cells. It is a key mediator of thrombin’s effects, and stimulation of PAR1 increases endothelial permeability, mitogenic responses, inflammation and fibrosis^4^,^23^,^31^,^32^. Previous work suggested that mesenchymal stem cells decrease oxidative stress, ER-stress response and TGF-β1 synthesis in alveolar epithelial cells exposed to bleomycin, through release of STC1^33^. However, the mechanism of STC1 actions remained to be defined. In the current work, we elucidated mechanisms of STC1-mediated protection from bleomycin-induced lung injury, and identified novel and hitherto unrecognized actions by STC1; i.e., inhibition of thrombin protein and activity; findings that are crucial for the design of future therapies for lung injury induced by radiation- or bleomycin.

Our study reveals increased thrombin protein abundance and activity in the lungs of WT mice after administration of bleomycin; however, thrombin abundance and activity were unchanged in the lungs of bleomycin-treated STC1 Tg mice. Since thrombin-induced and PAR1-mediated signaling increases the production of reactive oxygen species and ERK activation and inflammation in bleomycin-induced lung injury, we expected these to be mitigated by STC1. Indeed, compared with lungs of WT mice, lungs of STC1 Tg mice display lesser superoxide generation, ERK activation, production of cytokines/chemokines, and infiltration with lymphocytes, macrophages and neutrophils after bleomycin treatment.

What happens beyond PAR1 activation by thrombin? Current *in vitro* data in cultured pulmonary epithelial cells suggest that thrombin increases superoxide generation and activates ERK through PAR1-mediated pathway; STC1 attenuates baseline ERK activity, and blunts further activation of ERK by thrombin. Mechanistically, the actions of STC1 correlate with its effects on the redox state. Similar to paraquat (a superoxide generator), treatment of pulmonary epithelial cells with thrombin increases superoxide and ERK activity. And similar to MnTBAP (a cell permeable SOD mimic), treatment with STC1 diminishes superoxide level and inhibits thrombin-induced ERK1/2 activation. Importantly, STC1 fails to block thrombin-induced ERK1/2 activation in the presence of the superoxide generator paraquat. ERK phosphorylation and activation are redox sensitive^34^,^35^, and thrombin appears to increase ERK activity through increasing superoxide generation. Because STC1 suppresses superoxide generation, it provides a mechanism for inhibition of thrombin-mediated ERK1/2 activation downstream of PAR1. Thus, STC1 inhibits thrombin/PAR1-mediated ERK activation (signaling) through suppression of superoxide generation.

The regulation of thrombin activity by STC1 deserves further comment. A previous report suggested that activation of thrombin-activatable fibrinolysis inhibitor [(TAFI); an inhibitor of osteopontin (Spp1) and complement system components C3a & C5a] protects from acute lung injury^36^. Thus, inhibition of thrombin protein and activity as a mechanism for lung protection by STC1 is consistent with previous observations, and given the role of STC1 in diminishing thrombin-induced superoxide generation and ERK1/2 activation, our study hints at a potential innovative approach for the management of thrombotic conditions.

Survey of a broad range of cytokines/chemokines and growth factors (84 in total) in the lungs of bleomycin-treated mice identified a novel and hitherto unknown set of cytokines/chemokines that appear to play a role in the pathogenesis of bleomycin-induced pneumonitis. These include: Ccl17; Ccl2; Ccl7; Cxcl10; Cxcl16; Il18; Il1rn; Lif; Spp1 and Gusb. While thrombin-induced and PAR1-mediated expression of Ccl2 and CXcl10 has been reported^37^,^38^, and PAR1 has been implicated in Ccl7 expression in the setting of acute neutrophilic lung inflammation^32^, the involvement of Ccl17, Cxcl16, Il18, Il1rn, Lif and Spp1 in bleomycin-induced lung injury has not been recognized. Identification of these cytokines/chemokines by this study provides additional opportunities to examine mechanisms of bleomycin/thrombin/PAR1-induced lung injury/inflammation, and identifies potential therapeutic targets to ameliorate pneumonitis in bleomycin-treated patients. Importantly, STC1 blunts the expression of most of these cytokines/chemokines in the lung after bleomycin treatment.

In summary, current data identify STC1 as a novel inhibitor of bleomycin-induced lung injury. Protection by STC1 is mediated through diminishing thrombin protein level and activity, suppression of superoxide production, and subsequently inhibition of ERK1/2 activation and the expression of pro-inflammatory and pro-fibrotic cytokines/chemokines. STC1 is a naturally occurring protein, which exists in the serum at low concentrations; thus, delivery of STC1 before anticipated bleomycin administration may constitute a prophylactic approach to ameliorate bleomycin-induced lung injury. These findings broaden the array of potential therapeutic targets for the treatment of lung diseases characterized by inflammation and fibrosis.

## Additional Information

**How to cite this article**: Huang, L. *et al.* Stanniocalcin-1 inhibits thrombin-induced signaling and protects from bleomycin-induced lung injury. *Sci. Rep.* **5**, 18117; doi: 10.1038/srep18117 (2015).

## Figures and Tables

**Table 1:** Morphometric analysis of lung injury.

	WT Sham	STC1 Tg Sham	WT BLM	STC1 Tg BLM
IS	0	0	2 + 0.58	0.5 ± 0.29
P-value	NS	NS	*P < 0.01	^#^P < 0.05

H&E stained sections were viewed at ×200 magnification, and tissue injury grading was carried out as described in methods. The mean score from all examined fields was calculated as the inflammation score (IS). Means and ± SEMs are provided (n = 3–4). *P < 0.01, WT BLM vs WT Sham; ^#^P < 0.05, WT BLM vs STC1 Tg BLM; NS, WT Sham vs STC1 Tg Sham, or STC1 Tg Sham vs STC1 Tg BLM.

**Table 2:** Cytokine/chemokines that display increased expression in lungs of WT after bleomycin treatment, compared with lungs of saline-treated WT (red font in the “fold increase” column denotes fold upregulation relative to WT sham; n = 3).

Genes Over-Expressed in WT BLM vs. WT sham		
Gene Symbol	Fold increase	p-value
Ccl17	6.6295	0.003206
Ccl2	6.7706	0.016282
Ccl3	2.9236	0.081883
Ccl7	8.0755	0.002946
Cxcl10	4.6159	0.004979
Cxcl13	3.4948	0.215463
Cxcl16	5.1325	0.003251
Il18	3.0327	0.030913
Il1rn	3.4529	0.00796
Lif	3.0833	0.006896
Spp1	18.9586	0.005687
Gusb	2.2509	0.005652

**Table 3:** Comparison of cytokine/chemokine profile in the lungs of bleomycin-treated WT mice with bleomycin-treated STC1 Tg mice (red font in the “fold increase” column denotes fold upregulation relative to STC1 Tg BLM; n = 3).

Genes Over-Expressed in WT BLM vs. STC1Tg BLM		
Gene Symbol	Fold increase	p-value
Ccl17	2.0218	0.023863
Ccl19	2.0099	0.004189
Ccl2	2.99	0.033351
Ccl3	2.2521	0.219016
Ccl7	4.0391	0.005296
Cxcl10	2.2518	0.015744
Cxcl13	2.2942	0.309659
Cxcl16	2.8609	0.028026
Spp1	6.1254	0.011208
Tnfsf10	3.1896	0.064755
